# The role of frailty in the relationships between social relationships and health outcomes: a longitudinal study

**DOI:** 10.1186/s12889-024-18111-x

**Published:** 2024-02-24

**Authors:** Fereshteh Mehrabi, François Béland

**Affiliations:** 1 School of Public Health, Université de Montréal, Montréal, Québec, Canada; 2 Department of Psychology, Concordia University, Montréal, Québec, Canada; 3 Centre de recherche en santé publique (CReSP), Université de Montréal et CIUSSS du Centre-Sud-de-l’Île-de-Montréal, Montréal, Québec, Canada; 4 Lady Davis Institute for Medical Research, Jewish General Hospital, Montréal, Québec, Canada

**Keywords:** Social networks, Social participation, Social support, Depression, Chronic diseases, Cognitive function, Frailty, Moderation, Longitudinal, Ageing

## Abstract

**Background:**

Socially isolated older adults incur increased risks of adverse health outcomes, though the strength of this association is unclear. We examined whether changes in physical frailty moderated the associations between changes in social relationships and changes in health outcomes among older adults.

**Methods:**

This longitudinal study is based on three waves of the FRéLE study among 1643 Canadian community-dwelling older adults aged 65 years and older over 2 years. We performed latent growth curve modelling (LGMs) to assess changes with the assumption of missing not at random, adjusting for time-invariant covariates. We used the latent moderated structural equations (LMS) to test the interactions in LGMs. Social relationships were measured by social participation, social networks, and social support from different social ties. Frailty was assessed using the five components of the phenotype of frailty.

**Results:**

The results revealed that changes in frailty moderated changes in social participation (β = 3.229, 95% CI: 2.212, 4.245), social contact with friends (β = 4.980, 95% CI: 3.285, 6.675), and social support from friends (β = 2.406, 95% CI: 1.894, 2.917), children (β = 2.957, 95% CI: 1.932, 3.982), partner (β = 4.170, 95% CI: 3.036, 5.305) and extended family (β = 6.619, 95% CI: 2.309, 10.923) with changes in cognitive function and depressive symptoms, but not with chronic diseases. These results highlight the beneficial role of social relationships in declining depressive symptoms and improving cognitive health among older adults experiencing increases in frailty.

**Conclusions:**

The findings suggest that changes in social support have a protective and compensatory role in decreasing depressive symptoms and enhancing cognitive health among older adults with increasing frailty. Public health policy and strategies should consider the impact of social support on multiple health outcomes among older adults with increasing frailty. Further experimental studies and interventions are warranted to extend findings on the relationships between social relationships and health outcomes, targeting frail older adults. Future studies may also consider other health-related risk factors that may impact the associations between social relationships and health outcomes among older adults.

**Supplementary Information:**

The online version contains supplementary material available at 10.1186/s12889-024-18111-x.

## Background

Social isolation is a global public health concern with important implications for well-being in later life [1]. According to a recent systematic review and meta-analysis, the global prevalence of social isolation is about 15–25% among community-dwelling older adults [2]. Empirical research has indicated that social isolation is linked to poor physical, mental, and cognitive health outcomes in older age [3–5], rivalling the effects of cigarette smoking and obesity [3]. These risks are represented in an underpinning theoretical model proposed by Berkman and Krishna [6] that links structural (social networks and social participation) and functional (social support) aspects of social isolation to adverse health outcomes. Modelling risk factors for health outcomes can help us to better understand for whom social isolation may mostly impact various adverse health outcomes [7, 8].

Evidence has demonstrated that possible biological mechanisms such as frailty could explain the strength of the link between social isolation and health outcomes [9, 10]. Physical frailty is a state of increased vulnerability to external stressors due to a decline in physiological reserves across multiple organ systems [11, 12]. Frailty is associated with increased risks of disability, comorbidity [11], depression [13], cognitive impairment [14], and mortality [15]. Given the physiologic vulnerability inherent in physical frailty, it is plausible that the stress of isolation may result in adverse health outcomes in frail older adults compared to robust peers. The underlying mechanism is that social isolation is a stressor leading to poor health and challenges resilience, similar to the development of physical frailty due to the effect of stressors on physiological reserves [11, 16].

More specifically, social isolation may induce inflammation by influencing physiological responses to social and biological stressors [17, 18]. Therefore, someone experiencing social isolation may have a weakened immune system, lack the inflammatory response needed to recover from illness, and be more likely to be vulnerable to some diseases [16, 19]. Inflammatory processes may threaten the long-term health of older adults [10]. Likewise, frailty can manifest in older adults when such external stressors (e.g., acute illness, injury, or psychological stress) occur [20]. Relatedly, frail older adults have reduced stress tolerance due to decreased physiological reserves in the muscles, bones, and immune systems [21]. Fried and colleagues [11] provided support for the assumption that frail older adults are at greater risk for various deleterious outcomes due to key features of frailty such as muscle weakness, decreased endurance performance, and diminished physical activity.

Several prior studies and systematic reviews have demonstrated the link between social isolation and frailty [18, 22–24]. However, a paucity of research has examined the interplay between structural and functional aspects of social isolation, frailty, and health outcomes, and the results appear inconsistent among studies. The results of several cross-sectional studies illustrated that frailty was associated with disability, falls, and mortality, while social isolation was not related to health outcomes [25, 26]. Cross-sectional studies [27–29] and a longitudinal study [30] found that less social support was associated with frailty, cognitive decline, and falls; however, less participation in social activities was not linked to frailty and cognitive decline. None of these studies shed light on whether changes in one’s social networks are more or less problematic than changes in social support and social participation. The general conclusion derived from the existing evidence is that structural and functional aspects of social isolation may differently impact frailty and health outcomes among older adults.

In a longitudinal study examining the combined effects of frailty and social isolation on health outcomes, Hoogendijk and colleagues [31] illustrated that coexisting frailty and social isolation in older adults increased the risk of mortality compared to those with one or none of these conditions. However, Malini and colleagues [32] reported contradictory results that neither social support nor frailty was linked to fear of falling. Bevilacqua and colleagues [33] found that social isolation was related to chronic diseases but not frailty. These discrepancies suggest some degrees of uncertainty about the ability of frailty to alter the relationship between social isolation and health outcomes. Therefore, the hypothesis that frail and socially isolated older adults become more vulnerable to health-related conditions than their robust and isolated peers, needs further investigation.

**Fig. 1:**
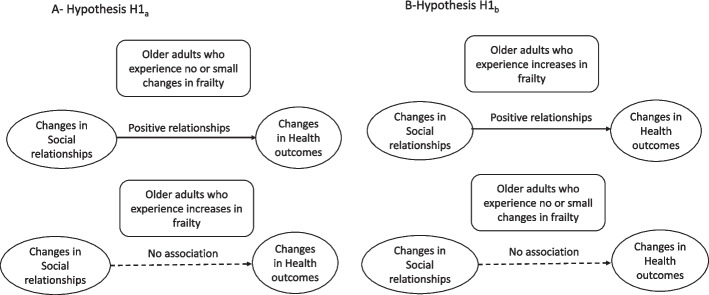
Model **a**) Greater changes in social relationships are linked to greater changes in health outcomes among older adults who experience no or small changes in frailty; however, changes in social relationships are not associated with changes in health outcomes among older adults who experience increases in frailty (Hypothesis H1a). Model **b**) Greater changes in social relationships are linked to greater changes in health outcomes among older adults who experience increases in frailty; however, changes in social relationships are not associated with changes in health outcomes among older adults who experience no changes in frailty (Hypothesis H1b)

To our knowledge and based on a recent scoping review [34], no studies have specifically examined the longitudinal moderating effects of frailty on the relationship between multidimensional social isolation and health outcomes. To address gaps and shortcomings in the literature, the objective of this study was to examine whether the relationship between changes in social relationships and changes in health outcomes varied based on changes in frailty among older adults. We proposed two alternative hypotheses that might explain the moderating role of frailty in this relationship (see Fig. 1).

H_1a_: Changes in social relationships will lead to changes in health outcomes among older adults who experience no or small changes in frailty compared to those who experience increases in frailty.

Rationale: Older adults with no or small changes in frailty have sufficient physiological reserves to mobilize social relationships. In contrast, the positive impact of changes in social relationships on changes in health outcomes will be small for older adults with increasing frailty because they lack the physiological reserves to benefit from social relationships.

H_1b_: Changes in social relationships will lead to changes in health outcomes among older adults with changes in frailty compared to those with no or small changes in frailty.

Rationale: Social relationships compensate for the lack of physiologic reserves in older adults with increasing frailty. Consequently, the beneficial effect of changes in social relationships on changes in health outcomes will occur in older adults with increasing frailty. However, the health of older adults with stable frailty will be less impacted by changes in social relationships as they need fewer social relationships to maintain or enhance their health status.

A null hypothesis is as follows:

- H_0_: Changes in frailty do not moderate the relationship between changes in social relationships and changes in health outcomes in older adults.

## Methods

### Study design and population

We analysed data from three waves of the FRéLE (Fragilité, une étude longitudinale de ses expressions/ Frailty: A longitudinal study of its expressions) population-based longitudinal study. Wave 1 of the study (baseline) took place in 2010, and subsequent data were collected yearly over two longitudinal waves (2011–2012). The FRéLE study was designed to have a significant number of men and women in each category of the phenotype of frailty. In order to estimate the sufficient sample size in each category of the phenotype of frailty, three different databases [35–37] were used. The results demonstrated that a sample stratified by sex (men and women) and age groups (65–74, 75–84, and 85 and over) with 270 participants in each of these subgroups was required to identify frailty trajectories over time [38, 39].

**Fig. 2:**
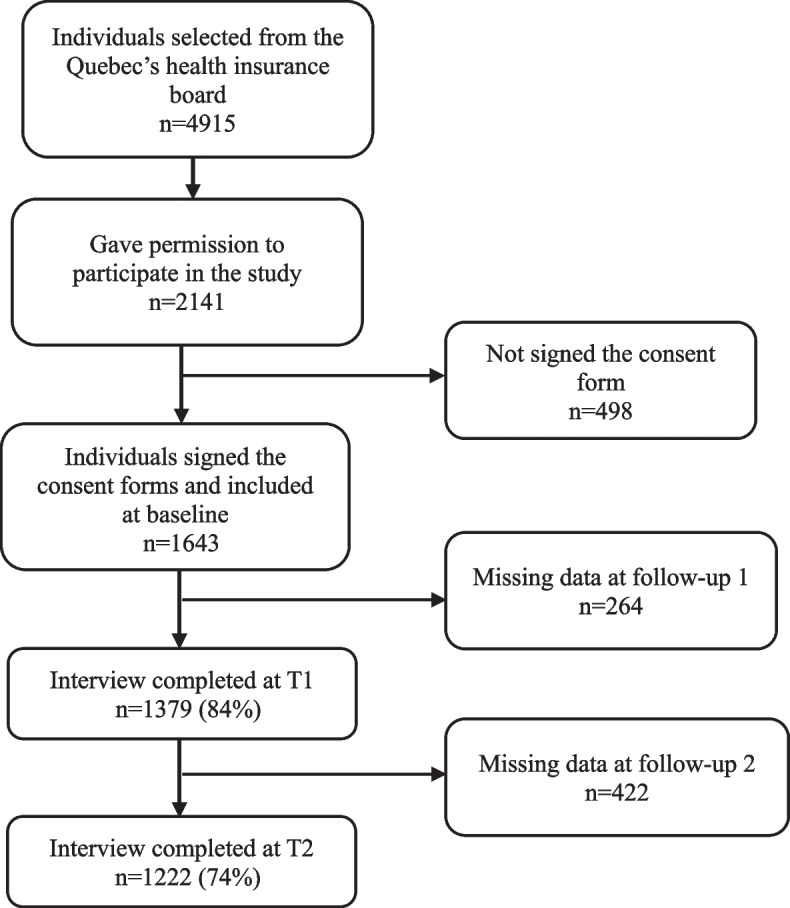
Flowchart of participants included in the FRéLE longitudinal study

The FRéLE sample was randomly selected from the Régie de l’Assurance Maladie du Québec (RAMQ)/Quebec’s health insurance board’s list. Participants were recruited from three areas in the province of Québec in Canada, including a metropolitan city (Montréal), a small city (Sherbrooke), and an urban-rural area (Victoriaville). A total of 4915 older adults were identified from the RAMQ’s list. The participants were eligible if they a) had no hearing impairment, b) were not admitted to a long-term care centre, c) were not hospitalized, d) had not participated in the Longitudinal Study on Nutrition and Successful Aging (NuAge) in Sherbrooke or Montréal, and e) were able to understand either English or French to answer the questionnaire during the interviews. No one with cognitive impairment was excluded. 2141 individuals were eligible and agreed to participate in the study. Finally, 1643 Community-dwelling older adults aged 65 and over signed a consent form and completed the questionnaires at baseline [38, 39] (see Fig. 2).

The sample was stratified by age (65–74;75–84; 85+), sex, and living areas. Twelve subgroups with an equal number of respondents were obtained. Of the 1643 participants at baseline, 84.4% participated in the first follow-up, and 88.4% of those from the first follow-up participated in the second follow-up. Losses were either due to mortality (13% over 2 years) or voluntary withdrawal and inability to contact (13% over 2 years).

In terms of the representativity of data, the FRéLE baseline results were compared with the Canadian Community Health Survey (CCHS) in the province of Québec. The results illustrated that the sociodemographic characteristics and health status of the FRéLE participants represented some characteristics of community-dwelling older adults across Québec. For example, 56.5% of participants in the FRéLE study had an education greater than high school compared to 55.1% of CCHS older respondents in Québec. Likewise, 48.4% of the FRéLE participants had an income higher than 30,000 CAD compared to 42.3% of Québec CCHS respondents [38, 39].

### Measures

#### Predictors: social relationships

According to Berkman’s [6] theory, we measured social relationships by social participation, social networks, and social support from different social ties, namely friends, nuclear family (i.e., children and spouse), and extended family (i.e., grandchildren and siblings). The Cronbach alpha coefficients of internal consistency for social variables are provided in Supplementary Table 1. The Cronbach alphas for social participation ranged from 0.69 to 0.66 across T0 to T2, indicating acceptable internal reliability. The Cronbach alphas for social networks with different types of social ties ranged from 0.70 to 0.88 across three time points, indicating acceptable and good scale reliability. Lastly, the Cronbach alpha estimates for social support from social ties ranged from 0.70 to 0.74 across three time points, demonstrating acceptable internal reliability in the current sample. Social participation, social networks, and social support were continuous variables.

#### Social participation

Social participation is measured by the CCHS - Healthy Aging [40]. The CCHS is a 12-item questionnaire scored on a five-point scale, ranging from 1 ("almost every day") to 5 ("never") [40]. The components of this scale included membership in community organizations, involvement in religious, community-based, and family activities, volunteering, playing music, painting, shopping, and going to restaurants, libraries, sports, and recreation centres. Scores were summed and higher scores indicated lower social participation. We reversed the score direction for consistency with social networks and support scales so that higher values represented a higher level of participation.

#### Social networks

We measured social networks with the longitudinal International Mobility in Aging Study’s (IMIAS) social network scale [41], a validated scale among older populations. Social networks comprised a series of questions asked separately about family members, friends, and children: “How many family/friends/ living children do you have?”; “How many of them do you see at least once a month?”; “How many of them do you have a very close relationship with?”; and “How many of them do you speak to by phone at least once a month?” Social contact with a spouse was not asked due to daily contact. The items for each social tie were summed to give a related social network score. The scores ranged from 1 ("never") to 5 ("always"), with greater scores indicating higher levels of social contact.

#### Social support

We used the IMIAS’s social support scale [41] to determine social support. The following questions were asked separately about one’s friends and members of one’s nuclear and extended family: “Do you help your family/ friends/ children/ partner from time to time?”; “Do you feel that you are loved by them?”; “Do they listen to you when you need to talk about your problems?”;” Do you feel that you play an important role in their lives?; and “Do you feel useful to them?” The scores ranged from 1 ("never") to 5 ("always"), with greater scores suggesting higher levels of social support.

#### The absence of social ties

Following the methodology proposed in the previous study [36], we created binary variables, indicating the absence of social ties. We assigned a score of zero to the participants with social ties (i.e., having friends) and a score of one to the participants without social ties (i.e., having no friends). The absence of social ties was a time-invariant variable as the number of participants’ social ties (e.g., children, siblings) did not often change in 2 years. In addition, we created a continuous variable for each social network and social support variable by multiplying each continuous social variable by its related binary variable (i.e., social networks with friends × no friends). We introduced these continuous variables along with binary variables simultaneously in the equations [36].

#### Moderator: frailty

In the FRéLE study, frailty was operationalized based on Fried’s [11] frailty criteria (More details about the operationalization of frailty are provided in supplementary files). The frailty scale consists of five components, including exhaustion, weight loss, low physical activity, slow gait, and low grip strength. Exhaustion was measured using a four-item measure of vitality from SF-36 [42] which was rated on a 5-point Likert scale, ranging from 0 to 100. Weight loss was measured by a self-reported unintentional weight loss of ≥4.5 kg during the past year [11]. Physical activity was measured with the Physical Activity Scale for the Elderly (PASE) [43, 44]. Gait speed was measured using a timed 15-ft (4572 m) walk. The threshold values were based on sex and standing height [45]. Handgrip strength was measured by the Martin Vigorimeter [46], using the mean of three trials for each hand (in kilopascals). Scores ranged from 0 to 400, with higher scores representing higher physical activity levels. Frailty refers to a clinical syndrome in the Fried [11] frailty phenotype. Unlike the frailty phenotype, we defined frailty as a marker and determinant of health outcomes based on the construct validity of frailty measurement assessed in the FRéLE study [47], which is consistent with the health-based conceptual frameworks of frailty proposed by Bergman and colleagues [48] and Gobben and colleagues [49]. Accordingly, we adopted Béland and colleagues’ [47] procedure and considered frailty a continuous latent variable. Higher scores equated to a lower level of frailty.

### Health outcomes

#### Cognitive health

Cognitive function was assessed using the Montreal Cognitive Assessment (MoCA), which has high reliability and internal consistency (α =0.83). Scores ranged from 0 to 30, with higher scores suggesting better cognitive performance (≥25) [50].

#### Comorbidity

Comorbidity was measured with the Functional Comorbidity Index (FCI) which is a validated scale for predicting physical function among older adults [51]. Diagnoses included 19 health problems (i.e., arthritis, asthma, heart disease, stroke, diabetes, visual and hearing impairment, obesity, cancer, etc.). Scores ranged from 1 to 19, with higher scores indicating comorbid conditions. The FCI was a continuous variable. In order to have consistency in health outcomes scales, we reversed the direction of this scale so that higher scores indicated less comorbidity.

#### Depressive symptoms

The 15-item Geriatric Depression Scale (GDS-15) was used to assess depressive symptoms. The GDS-15 was a continuous variable. The scores ranged from 0 to 15, with higher scores indicating higher depressive symptoms [52]. We reversed this scale so that higher scores indicated better mental health. The Cronbach alphas for the GDS were 0.75 in T0 and 0.78 in T1 and T2, indicating acceptable scale reliability.

#### Disability

We measured functional disability by the adapted Katz [53, 54] scale of Independence in Activities of Daily Living (ADLs) and the adapted Lawton [55, 56] scale of Instrumental ADLs (IADLs). ADLs consisted of bathing and showering, grooming, dressing, eating, toileting, walking across a room, getting in/out of bed, getting up from a chair, and cutting nails. IADLs were as follows: preparing hot meals, telephoning, using transportation, shopping, doing errands, light and heavy housekeeping, taking medications, and handling finances. A scale ranged from 0 to 9, with higher scores indicating greater functional limitations. As suggested by Spector and Fleishman [57], we combined ADLs and IADLs items into one single scale, representing a count variable.

#### Covariates

The time-invariant covariates comprised sociodemographic and life habit variables associated longitudinally with frailty [58] and health outcomes [59], including age (*65–98 years*), sex (*1 = female, 0 = male*), education levels (*range = 0–30, none -master/doctorate*), annual income (*range = 2500– > 80,000*), smoking status (*0 = non-smoker, 1 = former smoker, 2 = current smoker*), alcohol consumption (*1 = yes, 0 = no*), and sleeping disturbance (*1 = yes, 0 = no*). Age, education, and income were continuous variables.

### Statistical analysis

**Fig. 3:**
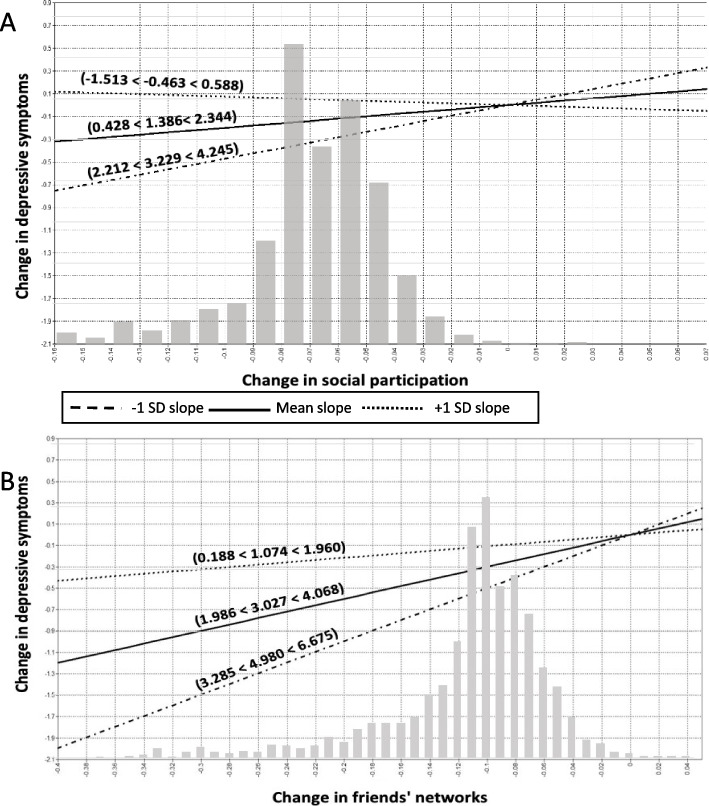
Interaction of changes in social participation and friends’ networks and depression with changes in frailty. Notes: For simplicity, random terms and covariates are not shown. Panel **A**: Changes in participation in social activities were positively associated with changes in decreasing depressive symptoms among individuals with negative changes in frailty (β = 3.229, 95% CI: 2.212, 4.245). However, this association was not significant for older adults with gradual and positive changes in frailty (β = − 0.463, 95% CI: − 1.513, 0.588). Panel **B**: Changes in contacts with friends were positively associated with changes in decreasing depressive symptoms among older adults with negative changes in frailty (β = 4.980, 95% CI: 3.285, 6.675)

**Fig. 4:**
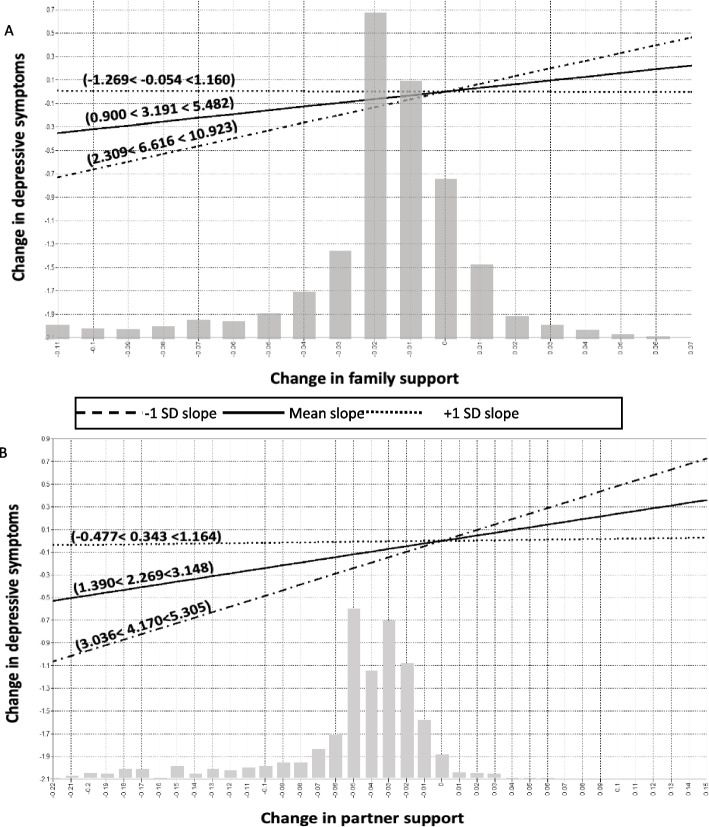
Interaction of changes in family and partner support and depression with changes in frailty. Notes: For simplicity, random terms and covariates are not shown. Panel **A**: Changes in social support from family members were positively associated with changes in decreasing depressive symptoms among individuals with negative changes in frailty (β = 6.619, 95% CI: 2.309, 10.923). However, this association was not significant for older adults with gradual and positive changes in frailty (β = − 0.054, 95% CI: − 1.269, 1.160). Panel **B**: Changes in social support from partner were positively associated with changes in decreasing depressive symptoms among individuals with negative changes in frailty (β = 4.170, 95% CI: 3.036, 5.305). However, this association was not significant for older adults with gradual and positive changes in frailty (β = − 0.343, 95% CI: − 0.477, 1.164)

**Fig. 5:**
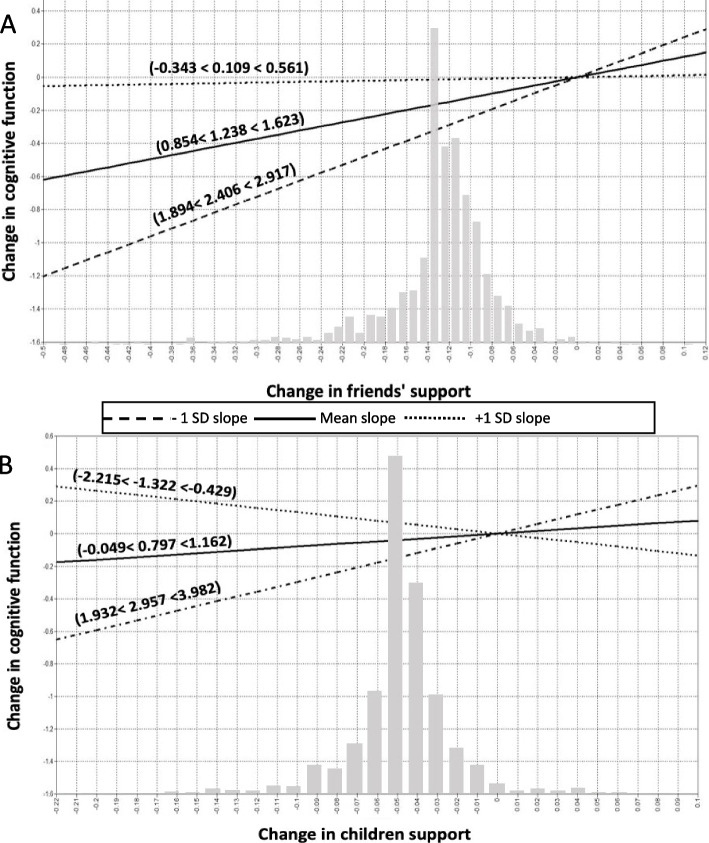
Interaction of changes in friends and children support and cognitive health with changes in frailty. Notes: For simplicity, random terms and covariates are not shown

We employed a series of latent growth curve models (LGMs) in Mplus [60] to assess changes, adjusting for time-invariant covariates. The LGMs estimated two indicators for each time-variant variable, including the initial status at baseline (intercept) and the growth change (slope). We estimated the interactions in LGMs using the latent moderated structural equations (LMS) approach under the normality assumption [61]. This approach minimizes the convergent problems and provides less biased estimates for coefficients and standard errors [61]. In this study, the distributions of all change scores were almost normal (see Figs. 3, 4 and 5). As the central aim of this study was to examine longitudinal associations, the interactions of slopes (indicating change over time) of social relationships and frailty on slopes of health outcomes were of primary interest. Interactions were conducted in the following steps. First, we regressed the slopes (changes) of health outcomes on the interactions between slopes (changes) of frailty and social relationships (continuous). Second, we regressed the slopes of health outcomes on the interactions between the slopes of frailty and the intercepts of social relationships (binary and continuous). Third, we regressed the slopes of health outcomes on the interactions between the intercepts of frailty and social relationships (binary and continuous). Fourth, we regressed the intercepts of health outcomes on the interactions between the intercepts of frailty and social relationships (binary and continuous) (Supplementary Fig. 1). Of note, the interactions involving intercepts in the third and fourth steps were not the subject of our moderation hypotheses and were added as control variables. Among predicted variables, the growth rate for disability was low and unstable. Therefore, we examined the intercept of disability, not the slope.

Estimation procedures for the interaction models are prone to convergence problems [62]. To minimize convergence problems, we estimated sets of starting values for residual variances and other terms from a collection of sub-models that were together approaching a saturated model [62]. In addition, convergence problems increased with an increasing number of interaction terms. Accordingly, we estimated LGMs separately for friends, nuclear family, extended family, and social participation, simultaneously entering all health outcomes into the models. We performed simple slope analyses [63] for significant interactions that depicted the association between changes in social isolation and health outcomes at one standard deviation (SD) below, one SD above, and at the mean value of changes in frailty. All continuous predictors and moderators were mean-centred. To test the significance of the interaction terms, we calculated *p*-values of the likelihood-ratio tests and compared models without and with interactions. We also used the Akaike Information Criterion (AIC), the Bayesian Information Criterion (BIC), and an adjusted BIC.

We compared the log-likelihood, number of parameters, and BIC values in all LGMs. The lower the BIC value, the better the model [64]. We estimated LGMs using the maximum likelihood estimator. The Poisson regression models were used for disability. The bootstrap procedure could not be applied in moderation analyses due to tedious computations. The estimations of interactions for social contacts with children and siblings were unstable, perhaps due to the small residual variances which were close to zero. Therefore, the findings were not reported. The number of missing data was 264 (16.1%) during the first follow-up (T1) and 421 (25%) during the second follow-up (T2). We handled missing data through a pattern mixture approach with the assumption of missing not at random [65]. The statistical significance level was defined at *p* < .05.

## Results

### Participants characteristics

Among 1643 participants at baseline, the average age was 78.7 years (SD = 7.9), and at least half were women (50.2%). Most of the participants were either former smokers (49%) or non-smokers (44%) and consumed alcohol (71%). More than half of the participants had no sleeping problems (58%). The averages for education and income levels ranged from 10.6 ± 4.7 and 4.1 ± 1.7 at baseline to 10.8 ± 4.6 and 4.3 ± 1.7 at T2, respectively (Supplementary Table 2). We compared participants who completed the study with those with missing values at follow-up. Those who dropped out were more likely to be women, frail, consume alcohol, and have chronic conditions and cognitive decline than those who remained in the study.

### Estimates of changes

Table 1 presents descriptive statistics on estimates of the average initial status and the average growth rate of variables of interest at population (fixed) and individual levels (random). At the population level, a variable may vary at different time-points. At the individual level, baseline averages and growth rates may head toward similar or different trends. High averages at baseline may be associated with downward rates of growth and low averages with upward rates of growth. At the individual level, all variables varied significantly at baseline as shown by averages and standard deviations (initial status). Growth rates were positive and significant for chronic conditions and disability, indicating a selective effect, such that respondents remaining in the sample were in better physical health than those who dropped out and the deceased. However, growth rates were not significant for depressive symptoms, cognitive function, and frailty. Nonetheless, their random terms were significant, indicating changes at the individual level. Individual growth rates did not vary significantly for disability. At the population level, average growth rates for all social relationships were negative and significant except for social contact with grandchildren (positive and significant), indicating an increase in social contact with grandchildren over time. At the individual level, only growth rates for social support from friends and spouse and social contact with grandchildren were significant.

**Table 1:** Parameters estimates from latent growth curve models

		Fixed			Random		
Health outcomes		Coef.	CI < 0.95	CI > 0.95	Coef.	CI < 0.95	CI > 0.95
Cognitive function	Average (i)	4.822^***^	4.773	4.867	0.669^***^	0.591	0.762
	Growth rate (s)	0.001	− 0.016	0.019	0.033^*^	0.002	0.070
	i WITH s				0.054^**^	0.017	0.090
Depressive symptoms	Average (i)	−1.330^***^	−1.402	−1.264	1.408^***^	1.229	1.600
	Growth rate (s)	0.002	−0.027	0.031	0.171^***^	0.100	0.238
	i WITH s				−0.110^*^	− 0.194	− 0.032
Chronic conditions	Average (i)	−1.584^***^	−1.641	− 1.528	0.879^***^	0.796	0.965
	Growth rate (s)	−0.076^***^	− 0.096	− 0.054	0.110^***^	0.070	0.149
	i WITH s				−0.008	− 0.047	0.031
Disability	Average (i)	−0.967^***^	−1.107	−0.828	2.730^***^	2.384	3.076
	Growth rate (s)	0.226 ^***^	0.156	0.297	0.001	−0.002	0.004
	i WITH s				−0.045	− 0.131	0.041
**Moderator**							
Frailty	Average (i)	0.225^***^	0.148	0.300	1.791^***^	1.657	1.910
	Growth rate (s)	0.001	−0.026	0.028	0.162^***^	0.106	0.208
	i WITH s				− 0.005	− 0.070	0.056
**Predictors**							
Social Participation	Average (i)	−7.022^***^	−7.073	−6.971	0.669^***^	0.607	0.737
	Growth rate (s)	−0.059^***^	− 0.078	− 0.040	0.014	− 0.014	0.041
	i WITH s				−0.011	− 0.042	0.019
Social Networks- Friends	Average (i)	0.921^***^	0.875	0.968	0.445^***^	0.373	0.517
	Growth rate (s)	−0.094^***^	−0.114	− 0.073	0.010	− 0.024	0.045
	i WITH s				−0.036	−0.080	0.007
Social Support- Friends	Average (i)	3.316^***^	3.233	3.396	2.004^***^	1.826	2.187
	Growth rate (s)	−0.116^***^	− 0.147	−0.088	0.100^**^	0.039	0.163
	i WITH s				−0.031	− 0.109	0.025
Social Networks-Children	Average (i)	1.905^***^	1.822	1.987	2.149^***^	1.962	2.373
	Growth rate (s)	−0.028^***^	− 0.041	− 0.016	0.022	− 0.018	0.065
	i WITH s				−0.083^***^	− 0.142	− 0.033
Social Support-Children	Average (i)	3.566 ^***^	3.473	3.649	2.402^***^	2.215	2.617
	Growth rate (s)	−0.037^***^	− 0.054	− 0.022	0.000	− 0.029	0.028
	i WITH s				−0.005	−0.036	0.026
Social Support-Partner	Average (i)	1.211^***^	1.148	1.274	1.212^***^	1.179	1.245
	Growth rate (s)	−0.040^***^	−0.053	−0.030	0.035^***^	0.019	0.049
	i WITH s				−0.036^***^	−0.055	− 0.019
Social Networks-Grandchildren	Average (i)	1.139^***^	1.1077	1.201	1.115^***^	1.007	1.223
	Growth rate (s)	0.018^**^	0.005	0.032	0.037^***^	0.024	0.049
	i WITH s				0.029^*^	0.000	0.058
Social Networks-Siblings	Average (i)	1.816^***^	1.731	1.899	1.953^***^	1.782	2.144
	Growth rate (s)	−0.066^***^	− 0.084	− 0.048	0.024	− 0.029	0.074
	i WITH s				−0.052	− 0.111	0.006
Social Support-Family	Average (i)	3.453^***^	3.403	3.503	0.600^***^	0.506	0.701
	Growth rate (s)	−0.030^**^	−0.052	−0.007	0.017	−0.021	0.054
	i WITH s				−0.015	−0.058	0.027

*Coef* coefficient, (i): intercept, (s): slope, “WITH” indicates covariance between intercept and slope, Number of Bootstrap Samples = 5000, ^*^ *p* ≤ 0,05, ^**^ *p* ≤ 0,01, ^***^ *p* ≤ 0,001. The models were unadjusted for covariates

### Moderating effects of changes in frailty on changes in social relationships and health

Multivariate LGMs revealed significant interactions between changes in social participation, contacts with friends, and support from different social ties and changes in frailty on changes in cognitive health and depressive symptoms. No other moderation effects were observed. Visualizing these interactions, Figs. 3, 4 and 5 illustrate the simple slope analyses of the conditional effects of changes in social relationships on changes in depressive symptoms and cognitive health across three levels of changes in frailty (average changes in frailty ±1 SD). To contextualize these changes, one standard deviation (SD) above the average change in frailty refers to positive changes in frailty among older adults (older adults with stable or lower levels of frailty), whereas one SD below the average change in frailty refers to negative changes in frailty (older adults who experienced increases in frailty).

Overall, 6 out of 24 interaction terms were significant after adjustment for covariates. The results of the simple slopes analyses demonstrated that greater changes toward social participation, support from friends, nuclear and extended family members, and contacts with friends were consistently and positively related to greater changes toward better cognitive health and fewer depressive symptoms among older adults with negative changes in frailty (1 SD below average) compared to those with average and positive (1 SD above average) changes in frailty (see Figs. 3, 4 and 5). However, the slope linking changes in social relationships to changes in depressive symptoms and cognitive health was almost flat or negative among older adults with positive changes in frailty. For example, as depicted in Fig. 5-Panel A, changes toward greater support from friends were positively associated with changes toward better cognitive function among individuals with negative changes in frailty (β = 2.406, 95% CI: 1.894, 2.917). However, this association was not significant for older adults with gradual and positive changes in frailty (β = 0.109, 95% CI: − 0.343, 0.561). Another example can be seen in Fig. 5-Panel B, where changes toward greater support from children were positively associated with changes toward better cognitive function among those with negative changes in frailty (β = 2.957, 95% CI: 1.932, 3.982). However, contrary to our hypotheses, changes toward greater social support from children were associated with changes toward decreasing cognitive function among older adults with positive changes in frailty (β = − 1.322, 95% CI: − 2.215, − 0.429). Of note, cases with decreasing change scores on frailty had lower scores on the frailty scales at baseline than cases with increasing change scores.

The grey bars in Figs. 3, 4 and 5 show the distributions of cases according to changes in social relationships. The distributions are almost normal with medians located at no change and the number of cases is decreasing with greater changes. The conditional effects of changes in social relationships on changes in cognitive health and depressive symptoms across changes in frailty appeared to be clustered among participants with decreasing loss of social relationships. In most cases, the interactions between changes in social relationships and frailty were significant in the extreme quartiles, indicating that the interaction effects were apparent for a few older adults. In particular, the effect size between changes in family support and changes in frailty was small (see Table 2).

**Table 2:** The effects of frailty on the association between social relationships and health outcomes

	Chronic conditions slope	Cognitive function slope	Depressive symptoms slope
Interaction effects	β [95%CI]	β [95%CI]	β [95%CI]
Social participation (T0) × Frailty (slope)	–	–	−0.347 [− 0.027, − 0.112]
Social participation (slope) × Frailty (slope)	–	–	− 17.577 [− 21.282, − 13.873]
Social networks-friends (slope) × Frailty (slope)	–	–	−15.022 [− 21.666, − 8.379]
Social support-friends (slope) × Frailty (slope)	–	− 8.833 [− 11.070, − 6.596]	–
Social support-children (slope) × Frailty (slope)	–	− 15.847 [− 19.225, − 12.469]	–
Social support-partner (slope) × Frailty (slope)	–	–	−16.639 [− 20.621, − 12.657]
Social support-family (slope) × Frailty (slope)	–	–	−25.657 [− 42.045, − 9.270]

Significant associations are solely presented. Hyphens (−) represent not-significant associations. All models were adjusted for covariates (e.g., age, sex, life habits, income, and education levels)

### Moderating effects of frailty on baseline social relationships

We found no interaction effects of baseline frailty and binary indicators of social relationships on baseline health outcomes, suggesting that the initial status of binary social variables was not part of the moderation terms with frailty. We found only two significant interactions involving continuous indicators of social relationships. Concordant with the second hypothesis (H_1b_), social participation at baseline was associated with changes toward decreasing depressive symptoms among older adults with negative changes in frailty (older adults with increasing frailty) (β = 0.059, 95% CI: 0.003, 0.116). However, contrary to our hypotheses and similar to Fig. 5-Panel B, baseline social participation was associated with changes toward increasing depressive symptoms among older adults with positive changes in frailty (β = − 0.056, 95% CI: − 0.107, − 0.004) (Supplementary Fig. 2). In line with the second hypothesis (H_1b_), social support from children was related to less functional limitations among frail older adults at baseline.

## Discussion

The link between social relationships and health is well-established, as demonstrated through Berkman and Krishna’s [6] theory and prior studies [1, 3]. However, the biological explanatory mechanisms by which social relationships connect to health, such as frailty, remain unknown. Our findings extend the research on the interplay between social relationships, frailty, and health in later life in three ways. First and foremost, in line with the second hypothesis (H_1b_), changes in frailty moderated the associations between changes toward increasing social participation, contacts with friends, and support from different social ties with changes toward better cognitive function and fewer depressive symptoms among older adults. The underlying generalised assumption to explain this effect is that social isolation is a stressful condition that may lead to serious health outcomes via inflammatory processes. Evidence suggests that social isolation can act as a stressor that may trigger an inflammatory response [10, 66]. The process is similar to frailty, a state of decreased physiological reserves and vulnerability to stressors, that can contribute to adverse health outcomes [11]. Therefore, the impact of social isolation on health outcomes may vary among older adults with low physiological reserves compared to those with a high level of physiological reserves. Older adults with stable or lower levels of frailty have sufficient physiological reserves and capacity to cope with challenges related to aging, respond to health stressors, and recover or maintain health status without support from others [67]. Therefore, social connection provides fewer benefits for health status among older adults with no or small changes in frailty compared to those who experienced increases in frailty. In this vein, the concept of physiological reserves buffers the positive impact of social relationships on health for older adults with stable frailty. However, social relationships compensate for age-related challenges among older adults with increasing frailty who have low physiological reserves to overcome stressors.

Second, we examined the distinct associations between multiple aspects of social relationships with health outcomes. This examination provided insight into the impacts of social relationships on various health outcomes and whether the effects of multidimensional social relationships on health differ based on frailty. The moderation results further corroborate two key points. First, changes in frailty moderated the longitudinal relationship between social relationships and depression symptoms and cognitive health, but not chronic diseases among older adults. Prior studies [68, 69] lend support to this assumption, reporting that perceived social relationships were linked to fewer depression symptoms rather than chronic diseases in later life.

Second, the moderation results elucidate the substantial role of social support from all types of social ties rather than social networks in reducing depressive symptoms in older adults with increasing frailty. In this view, meaningful and positive social interactions rather than regular contact with relatives or close friends appear to have health benefits [70, 71]. It is not the absence of social ties or frequency of social contacts – but the quality of those interactions – that has an important bearing on a person’s mental health [72]. The results are consistent with Berkman and Krishna’s [6] theory that the availability and supportiveness of social ties may explain the health-enhancing effects of social support. The underlying mechanism is that social support is a protective and compensatory factor against life stressors which may ameliorate vulnerability and lead to better health status among older adults with increasing frailty who experience physiological vulnerability to stressors [73, 74]. More importantly, supportive social ties may help reduce the impact of stress, improve physiological responses to stressors, and consequently enhance health outcomes among older adults with increasing frailty. According to Berkman and Krishna’s [6] theory, such social ties may provide essential emotional or instrumental support and companionship during illness by helping a person to better cope and compensate for physiological stress and recover more quickly from an illness.

Third, the moderation findings corroborate that higher levels of social activities at baseline and increasing changes in social participation compensated for a decline in mental status among older adults who experienced increases in frailty over 2 years. This result is in line with the findings from a previous longitudinal study [75], indicating that social gathering at baseline predicted changes in depressive symptoms among older adults over three waves, spanning 4 years. Likewise, another longitudinal study reported that social frailty was higher among physically frail older adults and was associated with depressive symptoms over time [76]. This result is generally concerning for age-friendly initiatives that focus predominantly on healthy individuals and leave behind people with health conditions and high-risk groups such as frail older populations [77].

This study has some limitations. We could not estimate changes in disability due to the low number of changes in the disability status over the two-year panel. We faced estimation problems for social contacts with children and siblings that limited our ability to estimate changes in these variables. We were also unable to simultaneously incorporate all social isolation variables in one model due to convergence issues. Another limitation is that the FRéLE study was conducted about 10 years ago and changes were observed in a short follow-up period (2 years). Accordingly, further analysis over a longer period would be valuable to capture changes in social relationships and health outcomes and unveil how differently these variables could be influenced by changes in frailty. Additionally, this study cannot rule out the possibility of reverse causation. The observational design of the study precludes any inference on causality, although time-varying variables were used. Future intervention research targeting contact with family members is necessary to clarify the directionality of our findings. Attrition is another limitation in the present study, resulting in a dropout rate of 25% and more healthy individuals remaining in the sample.

Despite these limitations, this study has several notable strengths. In addition to examining structural and functional aspects of social isolation, we considered whether different sources of social ties showed different patterns of association with multiple health outcomes in older age. Another strength of this study is the population-based longitudinal follow-up design with moderate sample size. Additionally, we employed comprehensive and validated measurements of social relationships, frailty, and health outcomes to capture different dimensions of social relationships and health status.

## Conclusions

In conclusion, this longitudinal study addresses one of the main components of healthy aging [78], underlining that the beneficial impact of social support and social participation on declining depressive symptoms mainly appeared among older adults with increasing frailty over time. However, social connection has limited benefits on the health status of older adults with a stable or lower level of frailty. It is thus of utmost importance to include and identify frail older adults with mental and cognitive conditions in social isolation interventions and programs. Public health policies and interventions should prioritize physically frail older populations in their programs and strategies to enhance the mental and cognitive well-being of older adults. Given that most older adults, particularly frail older adults, have experienced social isolation and loneliness due to the COVID-19 pandemic, there is some evidence to support targeting this vulnerable population in public health policies and programs. Future studies may consider other health-related risk factors (i.e., sedentary behaviours, sensory impairment) that may impact the relationships between social relationships and health outcomes among older adults. In addition, older adults with low socio-economic status and technology literacy, women, ethnic minorities, and indigenous communities may be particularly at risk for social isolation, frailty, and adverse health outcomes. Further studies should explore the interrelationships between social isolation, frailty, and health outcomes by gender, race, immigration status, and across different population subgroups. Fundamental questions remain about how public health policies may foster social programs to enhance social support and activity, targeting frail older people.

### Supplementary Information

**Supplementary Material.**

## Data Availability

The datasets used and/or analysed during the current study are available from the second author (FB) upon reasonable request.

## Abbreviations

ADL: Activities of daily living
AIC: Akaike information criterion
BIC: Bayesian information criterion
CCHS: Canadian community health survey
FCI: Functional comorbidity index
FRéLE: Fragilité, une étude longitudinale de ses expressions/Frailty: A longitudinal study of its expressions
GDS: Geriatric depression scale
IADL: Instrumental activities of daily living
IMIAS: International mobility in aging study
LGM: Latent growth curve models
LMS: Latent moderated structural methods
MoCA: Montreal cognitive assessment
PASE: Physical activity scale for the elderly
SD: Standard deviation
