# Correction: The role of frailty in the relationships between social relationships and health outcomes: a longitudinal study

**DOI:** 10.1186/s12889-024-18230-5

**Published:** 2024-03-13

**Authors:** Fereshteh Mehrabi, François Béland

**Affiliations:** 1 School of Public Health, Université de Montréal, Montréal, Québec Canada; 2 Department of Psychology, Concordia University, Montréal, Québec Canada; 3 School of Public Health, Université de Montréal, Montréal, Québec Canada; 4 Centre de recherche en santé publique (CReSP), Université de Montréal et CIUSSS du Centre-Sud-de- l’Île-de-Montréal, Montréal, Québec Canada; 5 Lady Davis Institute for Medical Research, Jewish General Hospital, Montréal, Québec Canada

BMC Public Health (2024) 24:602

10.1186/s12889-024-18111-x

Due to an error in the publication process 2 errors were present in the original publication of this article.

1. In Fig.2 the number of participants was obscured and not clearly visible

2. The caption for Fig.1 was incorrect, the incorrect and correct text is listed below.

**Incorrect**

Model (a) Greater changes in social relationships are linked to greater changes in health outcomes among older adults who experience increases in frailty; however, changes in social relationships are not associated with changes in health outcomes among older adults who experience no changes in frailty (Hypothesis H1a). Model (b) Greater changes in social relationships are linked to greater changes in health outcomes among older adults who experience no or small changes in frailty; however, changes in social relationships are not associated with changes in health outcomes among older adults who experience increases in frailty (Hypothesis H1b).

**Correct**

Model a) Greater changes in social relationships are linked to greater changes in health outcomes among older adults who experience no or small changes in frailty; however, changes in social relationships are not associated with changes in health outcomes among older adults who experience increases in frailty (Hypothesis H1a).

Model b) Greater changes in social relationships are linked to greater changes in health outcomes among older adults who experience increases in frailty; however, changes in social relationships are not associated with changes in health outcomes among older adults who experience no changes in frailty (Hypothesis H1b).

