# Supplementary material for: The role of frailty in the relationships between social relationships and health outcomes: a longitudinal study
Source: BMC Public Health. 2024 Feb 24;24:602. doi: 10.1186/s12889-024-18111-x (PMC10894481; doi:10.1186/s12889-024-18111-x)
Supplement: Supplementary file 1 — Supplementary Material. [file 12889_2024_18111_MOESM1_ESM.docx]

Supplementary files:

Supplementary Table 1. Cronbach alpha internal consistency estimates for social variables in three waves

| **Variables** | **T0** | **T1** | **T2** |
| --- | --- | --- | --- |
| Social participation | 0.69 | 0.67 | 0.66 |
| Social networks |  |  |  |
| Friends | 0.70 | 0.80 | 0.75 |
| Children | 0.87 | 0.90 | 0.88 |
| Siblings | 0.75 | 0.79 | 0.79 |
| Grandchildren | 0.74 | 0.71 | 0.71 |
| Social support |  |  |  |
| Friends | 0.72 | 0.67 | 0.65 |
| Children | 0.72 | 0.71 | 0.72 |
| Family | 0.70 | 0.73 | 0.72 |
| Partner /spouse | 0.73 | 0.74 | 0.74 |

Supplementary Figure 1. Latent growth curve moderation models

Notes: SR: Social Relationships; F: Frailty; HO: Health Outcomes. SRi: Intercept of social relationships (continuous variables); Fi: intercept of frailty; Binary SRi: Intercept of social relationships (binary variables); HOi: intercept of health outcomes; SRs: slope of social relationships (continuous variables); Fs: slope of frailty; HOs: slope of health outcomes; T0: time-point 0 (baseline, 2010); T1: time-point 1 (follow-up 1, 2011); T2: time-point 2 (follow-up 2, 2012). Continuous variables of social relationships are time-varying (T0–T2). Binary variables of social relationships are time-invariant (only at baseline (T0)). A correlation, indicated by the curved arrow between the intercept and slope, is included to account for the influence of initial scores on the growth trajectory. For clarity, covariates are not included. Different types of interactions are shown as follows:

**a and b)** The moderator effect of the intercept of frailty on the relationship between the intercepts of social relationships (continuous and binary variables) and health outcomes.

**c and d)** The moderator effect of the intercept of frailty on the relationship between the intercepts of social relationships (continuous and binary variables) and the slopes of health outcomes;

**e and f)** The moderator effect of the slope of frailty on the relationship between the intercepts of social relationships (continuous and binary variables) and the slopes of health outcomes;

1. The moderator effect of the slope of frailty on the relationship between the slope of social relationships (continuous variables) and the slopes of health outcomes.

Supplementary Table 2. Characteristics of the participants in three waves

| **Variables** | **Baseline (n=1643)** | **Time-point 1 (n=1386)** | **Time-point 2 (n=1224)** |
| --- | --- | --- | --- |
| **Covariates** |  |  |  |
| Age (years), mean ± SD | 78.7 ±7.9 | 78.1±7.8 | 77.6±7.6 |
| Gender (%) |  |  |  |
| Male | 49.8 | 49.1 | 48.1 |
| Female | 50.2 | 50.9 | 51.9 |
| Income, mean ± SD | 4.1 ± 1.7 | 4.2±1.7 | 4.3±1.7 |
| Education (years), mean ± SD | 10.6 ±4.7 | 10.7 ±4.7 | 10.8 ±4.6 |
| Smoking status (%) |  |  |  |
| Current smoker | 7.4 | 6.9 | 6.5 |
| Former smoker | 48.5 | 49.6 | 49.4 |
| Non-smoker | 44.1 | 43.5 | 44.1 |
| Alcohol consumption (%) |  |  |  |
| Yes | 71 | 71.1 | 71.8 |
| No | 29 | 28.9 | 28.2 |
| Sleeping disturbance (%) |  |  |  |
| Yes | 41 | 40.6 | 41.4 |
| No | 58.8 | 59.4 | 58.6 |
| **Predictors** |  |  |  |
| Social participation, mean ± SD | 42.8 ± 5.7 | 42.8±5.5 | 42.8±5.4 |
| Friends |  |  |  |
| Social Network, mean ± SD | 18 ± 16.6 | 15.6±15 | 15.2±15.3 |
| Social Support, mean ± SD | 16.1 ± 7.5 | 15.3±8.1 | 15.5±8.1 |
| No friends (%) | 14.8 | 14.1 | 14.1 |
| Nuclear family |  |  |  |
| Social Network-children, mean ± SD | 9.6 ± 7.4 | 9.3±7.1 | 9.2±6.8 |
| Social Support-children, mean ± SD | 17.5 ± 8 | 17.3±8 | 17.5±7.9 |
| Social Support-partner, mean ± SD | 11.9 ± 11.1 | 11.5±11 | 11.2±11 |
| No children (%) | 14.7 | 14.6 | 14 |
| No partner (%) | 45.5 | 46.2 | 46.7 |
| Extended family |  |  |  |
| Social Network-Grandchildren, mean ± SD | 11.3 ± 11 | 12±12.1 | 11.7±11.5 |
| Social Network- siblings, mean ± SD | 8.5 ±7.4 | 8.5±7.2 | 8.3±7.1 |
| Social support -family, mean ± SD | 17.1 ± 4.7 | 16.9±4.7 | 17±4.6 |
| No grandchildren (%) | 22.2 | 21.8 | 21.6 |
| No siblings (%) | 13 | 13.2 | 13.4 |
| **Moderator** |  |  |  |
| Frailty, mean ± SD | 20±8.6 | 21.4±9.1 | 21.2±9.3 |
| **Health outcomes** | | | |
| Disability, mean ± SD | 1.6±6.8 | 1.7±7.5 | 1.8±8.4 |
| Depressive symptoms, mean ± SD | 2.8±2.6 | 2.8±2.7 | 2.6±2.6 |
| Comorbidity, mean ± SD | 3.1±1.9 | 3.3±2.3 | 3.4±2.3 |
| Cognitive function, mean ± SD | 23.4±4.6 | 23.9±4.8 | 24±5.1 |
| Missing, % | 0 | 15.6 | 25.5 |

Notes: SD = Standard deviation, n=Total number.

Supplementary Table 3. Minimum and maximum values of variables in three waves

|  | **T0** | | **T1** | | **T2** | |
| --- | --- | --- | --- | --- | --- | --- |
| **Predictors** | **Min** | **Max** | **Min** | **Max** | **Min** | **Max** |
| Social participation | 22 | 55 | 23 | 55 | 25 | 55 |
| **Friends** | | | | | | |
| Social Networks | 0 | 70 | 0 | 60 | 0 | 70 |
| Social Support | 0 | 25 | 0 | 25 | 0 | 25 |
| **Nuclear family** | | | | | | |
| Social Networks-children | 0 | 38 | 0 | 36 | 0 | 30 |
| Social Support-children | 0 | 25 | 0 | 25 | 0 | 25 |
| Social Support-partner | 0 | 25 | 0 | 25 | 0 | 25 |
| **Extended family** | | | | | | |
| Social Networks -Grandchildren | 0 | 50 | 0 | 62 | 0 | 56 |
| Social Networks- siblings | 0 | 34 | 0 | 36 | 0 | 34 |
| Social support-family | 0 | 25 | 0 | 25 | 0 | 25 |
| **Moderator** |  |  |  |  |  |  |
| Frailty | 0 | 34 | 0 | 36 | 0 | 39 |
| **Outcomes** |  |  |  |  |  |  |
| Disability | 0 | 9 | 0 | 9 | 0 | 9 |
| Depression | 0 | 13 | 0 | 14 | 0 | 15 |
| Comorbidity | 0 | 7 | 0 | 13 | 0 | 12 |
| Cognitive function | 0 | 30 | 0 | 30 | 0 | 30 |

Notes: Due to the large variances, we rescaled the scores by dividing them by a constant of such size that their variances fall between one and ten after rescaling (Muthén & Muthén, 2017). We divided each score in Mplus as follows: Social support and cognitive decline/5; frailty/6; social network-siblings and children/5; social networks-grandchildren/10; social networks-friends/20; social participation/6; depression and chronic diseases/2.

Supplementary Figure 2. Relationship between baseline social participation and change in depressive symptoms as a function of change in frailty (mean slope ± 1 SD).

**Operationalization of frailty**

The controversial debate is about whether frailty is a medical syndrome or not. Bandeen-Roche et al. (2006) performed a latent class analysis (LCA) to examine the internal validity of the phenotype of frailty and test the degree to which the five criteria of frailty aggregate into a clinical syndrome, using the data from the Women’s Health and Aging Studies (WHAS) I and II. Frailty is considered a syndrome if there are two classes or more. Bandeen-Roche et al. (2006) identified three classes and the prevalence of each criterion of frailty increased across classes which suggested an increase in overall frailty status. The results support the internal validity of the phenotype of frailty, identify frailty as a clinical syndrome, and justify the three categories of frailty status (frail, prefrail, and robust) (Bandeen-Roche et al., 2006).

In line with the WHAS study, Béland et al. (2020) evaluated the construct validity of Fried’s frailty phenotype in the FRéLE study. Similar to the WHAS study, Béland et al. (2020) performed the LCA to examine if the frailty’s criteria aggregated into a clinical syndrome. According to the Bandeen-Roche et al. (2006) procedure, Béland et al. binarized the five criteria of frailty and compared the LCA results with the three identified frailty classes in the WHAS study. The results of the FRéLE study were similar to the WHAS study and thereby Béland et al. (2020) rejected the hypothesis of homogeneity. This indicates that the FRéLE study’s sample is an acceptable starting point to test the validity of the frailty phenotype scale. In addition to the one-class model null hypothesis in the WHAS study, Béland et al. (2020) tested the null hypothesis of more than one-class in the FRéLE study, using a continuous scale of Fried’s phenotype of frailty (2001). The results have shown that individuals were not heterogeneous between classes and therefore, frailty is not defined as a clinical syndrome but as a marker of health status in the FRéLE study.

Frailty is defined as a marker of health status in the current study based on the Béland et al. study (2020) which is also consistent with the health-based conceptual frameworks of frailty proposed by Bergman et al. (2004) and Gobben et al. (2010). Based on this specific feature of frailty as health status, we were able to examine the moderator effects of frailty on the relationships between social relationships and health outcomes. Accordingly, frailty as one of the determinants of health status could manifest in the relationships between social relationships and health outcomes among older adults.

**References**

Bandeen-Roche, K., Xue, Q.-L., Ferrucci, L., Walston, J., Guralnik, J. M., Chaves, P., Zeger, S. L., & Fried, L. P. (2006). Phenotype of frailty: characterization in the women's health and aging studies. *The Journals of Gerontology Series A: Biological Sciences and Medical Sciences*, *61*(3), 262-266.

Béland, F., Julien, D., Wolfson, C., Bergman, H., Gaudreau, P., Galand, C., Fletcher, J., Zunzunegui, M.-V., Shatenstein, B., & Kergoat, M.-J. (2020). Revisiting the hypothesis of syndromic frailty: a cross-sectional study of the structural validity of the frailty phenotype. *BMC geriatrics*, *20*(1), 1-13.

Bergman, H., Béland, F., Karunananthan, S., Hummel, S., Hogan, D., & Wolfson, C. (2004). Developing a Working Framework for Understanding Frailty Howard Bergman, MD. *Gérontologie et société*, *109*, 15-29.

Fried, L. P., Tangen, C. M., Walston, J., Newman, A. B., Hirsch, C., Gottdiener, J., Seeman, T., Tracy, R., Kop, W. J., & Burke, G. (2001). Frailty in older adults: evidence for a phenotype. *The Journals of Gerontology Series A: Biological Sciences and Medical Sciences*, *56*(3), M146-M157.

Gobbens, R., Luijkx, K., Wijnen-Sponselee, M. T., & Schols, J. (2010). Towards an integral conceptual model of frailty. *The journal of nutrition, health & aging*, *14*(3), 175-181.

Muthén, L. K., & Muthén, B. (2017). *Mplus user's guide: Statistical analysis with latent variables, user's guide*. Muthén & Muthén.
